# The impact of poor asthma control among asthma patients treated with inhaled corticosteroids plus long-acting β_2_-agonists in the United Kingdom: a cross-sectional analysis

**DOI:** 10.1038/s41533-017-0014-1

**Published:** 2017-03-09

**Authors:** Ian D. Pavord, Nicola Mathieson, Anna Scowcroft, Riccardo Pedersini, Gina Isherwood, David Price

**Affiliations:** 1 Respiratory Medicine Unit, Nuffield Department of Medicine, NDM Research Building, Old Road Campus, University of Oxford, Oxford, OX3 7FZ UK; 2 Boehringer Ingelheim Limited, Ellesfield Avenue, Bracknell, Berkshire RG12 8YS UK; 3 Kantar Health, Health Outcomes Practice, 19-31 Church Street, Epsom, Surrey KT17 4PF UK; 4 RTI Health Solutions, Avinguda Diagonal, 605, 9-1, Barcelona, 08028 Spain; 5 Academic Primary Care, University of Aberdeen, Polwarth Building, Foresterhill, Aberdeen, AB25 2ZD UK

## Abstract

There are several new treatment options for patients whose asthma remains uncontrolled on free-dose and fixed-dose combinations of inhaled corticosteroids plus long-acting β_2_-agonists (ICS+LABA). In order to evaluate the likely impact of these treatments, we assessed the effect of uncontrolled asthma on healthcare and patient burden within the UK among adult patients treated with ICS+LABA. Data obtained from 2010–2011 UK National Health and Wellness Surveys identified 701 patients treated with ICS+LABA. Patients with not well-controlled asthma (Asthma Control Test™ score <20) were compared with well-controlled asthma (score ≥ 20) patients on multiple measures. Cost burden was calculated using healthcare resource utilisation models and work productivity and impairment questionnaire. Overall, 452 and 249 patients reported not well-controlled and well-controlled asthma, respectively. A greater proportion of not well-controlled patients visited the accident & emergency department (21 vs. 14%, *P* = 0.016), were hospitalised (13 vs. 8%, *P* = 0.022) and had lower mental and physical health-related quality of life (*P* < 0.001) and impaired work productivity and activity scores: presenteeism (23 vs. 11%, *P* < 0.001), work impairment (29 vs. 17%, *P* < 0.001) and activity impairment (46 vs. 24%, *P* < 0.001). Calculated direct and indirect yearly costs/person doubled among not well-controlled compared to well-controlled asthma patients (£6592 vs. £3220). Total cost to society was estimated at £6172 million/year (direct costs, £1307 million; indirect costs, £4865 million). In conclusion, not well-controlled asthma is common among UK adults treated with ICS+LABA, resulting in impairments across a number of important health outcomes and represents a significant unmet need and resource burden.

## Asthma: Drug combo leaves many with uncontrolled disease

Many people who take inhaled steroids combined with long-acting β2-agonist drugs still have poorly controlled asthma. A team led by Ian Pavord from the University of Oxford, UK, identified 701 people from the 2010–2011 UK National Health and Wellness Surveys who were taking this drug combination for their asthma. The researchers found that nearly two-thirds of these individuals had poorly controlled asthma associated with more visits to the emergency room, worse quality of life (both mentally and physically), impaired productivity and other health problems. The calculated direct and indirect costs per person with poorly controlled asthma were about double that for someone whose asthma was under control. The authors conclude that better treatment and management is needed to reduce costs and address the unmet medical need for people with persistent uncontrolled asthma.

## Introduction

International strategies set forth by the Global Initiative for Asthma (GINA), as well as UK-specific recommendations and guidelines help in effective clinical management.^1, 2^ Inhaled corticosteroids (ICS) are the first choice agent for patients requiring regular maintenance treatment.^3^ Despite the presence of established treatment guidelines, many asthma patients still experience persistent symptoms and, therefore, poor disease control.^4^

For patients who experience persistent symptoms or exacerbations, guidelines advise the stepwise use of higher doses of ICS and/or the use of additional adjunct therapies such as long-acting β_2_-agonists (LABAs).^1^ ICS and LABA fixed-dose combinations are widely used and have been estimated to account for approximately 54% of all ICS prescribed in the UK and represent 80% of ICS prescribing costs.^5^

When asthma is not well-controlled, patients generally experience functional limitations and are at an increased risk of exacerbations, pulmonary function loss and mortality resulting in significant direct and indirect resource costs.^6–9^ New treatment options, including tiotropium and biological agents, are available to treat these patients.^10^ A clear understanding of these costs is important in order to assess the impact of these therapies.

Several studies looking at direct costs have been conducted in the UK: a study of over 12,000 patients found that poorly controlled asthma increased the risk of exacerbations and the need for emergency medical attention, which was in turn associated with a three-fold to four-fold increase in care costs.^11, 12^ A 2013 study estimated the total direct cost of treating asthma in the UK to be over £750 million, with the individuals with uncontrolled asthma and multiple exacerbations (~ 2.7%) accounting for nearly £53 million of care costs (~ 7%).^13^

Indirect costs associated with poorly controlled asthma are also recognised to be significant.^14^ They can include costs arising from impaired work and education, productivity and absenteeism, transportation to and from medical visits, and impairments in quality of life.^15–18^ European studies have reported significant indirect costs of up to €1800 per trimester in Spanish patients with not well-controlled asthma,^19^ underscoring the importance of effectively addressing inadequate control.^20^

Despite an increasing body of real-world evidence demonstrating a link between control of asthma symptoms and the associated burden,^21–23^ there remains a paucity of data on the burden of uncontrolled asthma on patients treated specifically with ICS and LABA combinations. An enhanced understanding of the direct and indirect costs associated with asthma control among this patient group is needed to better inform treatment guidelines and scientific research. This study aimed to investigate the impact of uncontrolled asthma among adult patients treated with ICS+LABA on healthcare resource and patient burden based on data obtained from the 2010 and 2011 UK National Health and Wellness Surveys (NHWS).

## Results

### Descriptive analyses

The combined 2010–2011 NHWS sample of the UK adult respondents self-reporting a physician diagnosis of asthma (*N* = 3105) projected to the overall 2011 UK adult population yielded an estimate of 5,088,605 asthma patients, corresponding to 10.4% of the adult population (Supplementary Table 1 provides additional information on age and gender). This estimate was higher than the Quality and Outcomes Framework figures,^24^ although its 5.9% prevalence of asthma for 2011–2012 in the UK was derived from data that were not weighted by age and gender. Among the 3105 surveyed asthma respondents (Supplementary Table 2), almost 30% reported currently being treated with ICS and LABA in free-dose combination or fixed-dose combination (ICS+LABA). Of these, approximately 20% self-reported a concomitant physician diagnosis of chronic obstructive pulmonary disease (COPD), chronic bronchitis or emphysema (*n* = 880, projected to 1,430,634) and were therefore excluded from further analyses. The remaining 80% (*n* = 701, projected to 1,142,033) were divided into two groups according to the Asthma Control Test (ACT) score: 36% (*n* = 249, projected to 402,293) had well-controlled asthma and 64% (*n* = 452, projected to 739,740) had not well-controlled asthma.

### Bivariate analyses

Tables 1–4 describe the personal characteristics, health outcomes, asthma characteristics and adherence of those with well-controlled and not well-controlled asthma. Not well-controlled asthma patients tended to have lower income (48 vs. 31% earn less than £20,000), lower levels of employment (42 vs. 53%) and a greater proportion of obesity (38 vs. 25%), while well-controlled asthma patients were more likely to be overweight (42 vs. 31%; Table 1).

**Table 1:** Comparison of asthma respondents with well-controlled and not well-controlled asthma treated with ICS+LABA without concurrent diagnosis of COPD, chronic bronchitis, or emphysema: personal characteristics

ICS+LABA (free and fixed-dose combination)							
No COPD diagnosis	Not well-controlled		Well-controlled		Total		Test
	*n*	Index	*n*	Index	*n*	Index	*P*
*Gender*							0.263
Female	288	63.7%	148	59.4%	436	62.2%	
*Age, years*							0.812
*n*, mean	452	48.9	249	48.6	701	48.8	
*Married or living with partner*							0.242
No	317	70.1%	185	74.3%	502	71.6%	
*Education*							0.056
Less than university graduate	232	51.3%	109	43.8%	341	48.6%	
*Annual household income*							<0.001
Less than £10,000	73	16.2%	27	10.8%	100	14.3%	
£10,000 to £19,999	142	31.4%	50	20.1%	192	27.4%	
£20,000 to £49,999	163	36.1%	105	42.2%	268	38.2%	
£50,000 or more	30	6.6%	34	13.7%	64	9.1%	
Declined to answer	44	9.7%	33	13.3%	77	11.0%	
*Employment status*							0.004
Not employed	263	58.2%	117	47.0%	380	54.2%	
*BMI, kg/m* ^*2*^							0.002
Underweight or normal range (BMI < 25)	118	26.1%	66	26.5%	184	26.2%	
Overweight (25 ≤ BMI < 30)	138	30.5%	105	42.2%	243	34.7%	
Obese (BMI ≥ 30)	171	37.8%	62	24.9%	233	33.2%	
Declined to answer	25	5.5%	16	6.4%	41	5.9%	
*Smoking behaviour*							<0.001
Not smoking	367	81.2%	229	92.0%	596	85.0%	
*Charlson Comorbidity Index*							0.273
*n*, mean	452	0.31	249	0.25	701	0.29	
*Sample size*	452		249		701		

*BMI* body mass index, *COPD* chronic obstructive pulmonary disease, *ICS* inhaled corticosteroids, *LABA* long-acting β_2_-agonist

**Table 2:** Comparison of asthma respondents with well-controlled and not well-controlled asthma treated with ICS+LABA without concurrent diagnosis of COPD, chronic bronchitis, or emphysema: health outcomes

ICS+LABA (free and fixed-dose combination)							
No COPD diagnosis	Not well-controlled		Well-controlled		Total		Test
	*n*	Mean	*n*	Mean	*n*	Mean	*P*
*Quality of life (SF-12v2)*							
Mental component score	452	43.0	249	47.4	701	44.6	<0.001
Physical component score	452	39.9	249	48.1	701	42.8	<0.001
Health utility	452	0.65	249	0.74	701	0.69	<0.001
*Work productivity and activity impairment*							
Employed only							
Absenteeism (%)	175	9.2	126	6.8	301	8.2	0.394
Presenteeism (%)	165	23.2	119	11.0	284	18.1	<0.001
Overall work impairment (%)	175	29.2	126	16.8	301	24.0	<0.001
Employed *+* unemployed							
Activity impairment (%)	452	46.2	249	24.2	701	38.4	<0.001
*Use of healthcare resources (not limited to asthma), past 6 months*							
Visited a physician (n, %)							0.053
GP	62	13.7%	35	14.1%	97	13.8%	
Specialist	369	81.6%	191	76.7%	560	79.9%	
None of the above	21	4.7%	23	9.2%	44	6.3%	
A&E department visits (n, %)							0.016
No	355	78.5%	214	85.9%	569	81.2%	
Yes	97	21.5%	35	14.1%	132	18.8%	
Hospitalisations (n, %)							0.022
No	394	87.2%	231	92.8%	625	89.2%	
Yes	58	12.8%	18	7.2%	76	10.8%	
Number of GP visits	452	3.6	249	2.3	701	3.1	<0.001
Number of specialist visits	452	4.1	249	2.8	701	3.6	0.002
Number of A&E department visits	452	0.13	249	0.07	701	0.11	0.022
Number of hospitalisations	452	0.22	249	0.14	701	0.19	0.016
*Sample size*	452		249		701		

*A&E* accident & emergency, *COPD* chronic obstructive pulmonary disease, *GP* general practitioner, *ICS* inhaled corticosteroids, *LABA* long-acting β_2_-agonist, SF-12v2; Medical outcomes study 12-item short form survey instrument

**Table 3:** Comparison of asthma respondents with well-controlled and not well-controlled asthma treated with ICS+LABA without concurrent diagnosis of COPD, chronic bronchitis, or emphysema: asthma characteristics

ICS+LABA (free and fixed-dose combination)							
No COPD diagnosis	Not well- controlled		Well-controlled		Total		Test
	*n*	Index	*n*	Index	*n*	Index	*P*
*Length of diagnosis (years)*							0.432
*n*, mean	450	22.5	247	21.6	697	22.2	
*ACT score*							<0.001
*n*, mean	452	15.3	249	22.8	701	17.9	
*Asthma control, past 4 weeks*							<0.001
*n*, mean	452	3.4	249	4.5	701	3.8	
*Talked to doctor about their asthma, past 12 months*							<0.001
*n*, mean	450	2.7	247	1.3	697	2.2	
*Frequency of asthma problems*							<0.001
Daily	189	41.8%	37	14.9%	226	32.2%	
4–6 times a week	72	15.9%	10	4.0%	82	11.7%	
2–3 times a week	97	21.5%	28	11.2%	125	17.8%	
Once a week	28	6.2%	25	10.0%	53	7.6%	
2–3 times a month	38	8.4%	41	16.5%	79	11.3%	
Once a month or less often	28	6.2%	108	43.4%	136	19.4%	
*Self-reported asthma severity when taking medication*							<0.001
Mild intermittent	187	41.4%	212	85.1%	399	56.9%	
Mild persistent	168	37.2%	30	12.1%	198	28.3%	
Moderate persistent	90	19.9%	7	2.8%	97	13.8%	
Severe persistent	7	1.6%	0	0.0%	7	1.0%	
*Self-reported asthma severity when not taking medication*							<0.001
Mild intermittent	21	4.7%	50	20.1%	71	10.1%	
Mild persistent	74	16.4%	82	32.9%	156	22.3%	
Moderate persistent	180	39.8%	75	30.1%	255	36.4%	
Severe persistent	177	39.2%	42	16.9%	219	31.2%	
*Sample size*	452		249		701		

*ACT* asthma control test™, *COPD* chronic obstructive pulmonary disease, *GP* general practitioner, *ICS* inhaled corticosteroids, *LABA* long-acting β_2_-agonist

**Table 4:** Comparison of asthma respondents with well-controlled and not well-controlled asthma treated with ICS+LABA without concurrent diagnosis of COPD, chronic bronchitis, or emphysema: adherence

ICS+LABA (free and fixed-dose combination)							
No COPD diagnosis	Not well-controlled		Well-controlled		Total		Test
	*n*	Index	*n*	Index	*n*	Index	*P*
*Adherence score (MGLS)*							0.147
*n*, mean	452	0.94	249	0.81	701	0.89	
*Adherence level (MGLS)*							0.361
High	224	49.6%	137	55.0%	361	51.5%	
Medium	169	37.4%	85	34.1%	254	36.2%	
Low	59	13.1%	27	10.8%	86	12.3%	
*Sample size*	452		249		701		

*COPD* chronic obstructive pulmonary disease, *ICS* inhaled corticosteroids, *LABA* long-acting β_2_-agonist, MGLS, Morisky, Green, and Levine scale

A greater proportion of not well-controlled asthma patients visited the Accident & Emergency (A&E) (21 vs. 14%) or were hospitalised (13 vs. 8%) in the previous 6 months, saw their general practitioner (GP) or specialist more often (8 vs. 5 times in previous 6 months), talked to their doctor about their asthma more often (on average 3 vs. 1 time over the past 12 months) and had more severe asthma symptoms regardless of whether they were taking their asthma medication or not (Tables 2 and 3). These healthcare visits were not necessarily asthma related, but could be due to any cause. The health-related quality of life (HRQoL) of not well-controlled asthma patients was lower on all three summary scores of the Short Form Survey Instrument (SF-12v2) (mental component summary [MCS]: 43 vs. 47; physical component summary [PCS]: 40 vs. 48; health utility: 0.65 vs. 0.74), while their work and activity impairments were greater: presenteeism (23 vs. 11%), overall work impairment (29 vs. 17%) and activity impairment (46 vs. 24%).

There was no difference in reported adherence using the Morisky, Green, and Levine scale (MGLS; Table 4), suggesting that their diminished asthma control may be due to other reasons such as incorrect choice of inhaler, poor inhalation technique, smoking, comorbidity^25^ or worsening of their condition.

### Multivariate analyses

#### Health-related quality of life (SF-12v2)

Not well-controlled asthma patients had worse HRQoL compared with well-controlled patients on all three metrics (MCS, PCS and health utility; Supplementary Table 3a). It also appears that MCS scores improved with age and decreased in people who smoked and among people with more comorbidities as measured by the Charlson Comorbidity Index (CCI). PCS scores, on the other hand, decreased with age, as well as with obesity and CCI. Finally, health utility was lower in males, obese respondents, smokers and those with more comorbidities. All effects were significant (Supplementary Table 3).

#### Adherence (MGLS)

The overall MMAS score did not show any significant difference between both sets of patients. Instead, adherence (overall MGLS score) seemed to be affected by gender, age and income (Supplementary Table 3b): males had a lower likelihood of being non-adherent, and being older and having a higher income were associated with a higher likelihood of being non-adherent.

#### Use of healthcare resources

Supplementary Table 4a shows that use of healthcare resources was significantly higher among patients with poorly controlled asthma (number of visits to GP, specialist, A&E and hospital), and increased with comorbidities (CCI). In addition, adherence to prescribed asthma medication was associated with significantly more specialist and A&E visits, suggesting that patients with more severe asthma may be more adherent to their asthma medications.

#### Work productivity loss (Work productivity and activity impairment [WPAI])

Absenteeism, presenteeism, overall work impairment and activity impairment were all higher in not well-controlled asthma patients compared to those with well-controlled asthma (Supplementary Table 4b). When combined, all scores were lower among males; absenteeism decreased with adherence; higher annual household income was associated with higher absenteeism, overall work impairment and activity impairment; age and non-adherence were positively associated with absenteeism and higher body mass index was associated with higher absenteeism and presenteeism.

#### Cost analysis

Supplementary Table 5 and Table 5 summarise the yearly costs per person, which were doubled among not well-controlled asthma patients compared with well-controlled patients (£6592 vs. £3220). Projected yearly costs for the 2011 UK adult population treated with ICS+LABA were almost four times higher for the not well-controlled group (£4877 million vs. £1295 million). The total combined cost to society was estimated at £6172 million per year, where direct costs amount to £1307 million and indirect costs to £4865 million.

**Table 5:** Cost analysis: adjusted means and projections to the 2011 adult population of UK treated with ICS+LABA

Yearly costs	Not well-controlled		Well-controlled	
	£ per person	Weighted total (m)	£ per person	Weighted total (m)
Physician visits	551	408 m	375	151 m
A&E department visits	95	71 m	60	24 m
Hospitalisations	708	524 m	322	130 m
Direct costs	1355	1002 m	758	305 m
Absenteeism	2747	2032 m	1012	407 m
Presenteeism	4480	3314 m	2181	877 m
Indirect costs (overall work impairment)	5238	3874 m	2463	991 m
Total costs	6592	4877 m	3220	1295 m

*A&E* accident & emergency, *ICS* inhaled corticosteroids, *LABA* long-acting β_2_-agonist, *m* million

## Discussion

### Main findings

The current study sought to quantify the burden of poorly controlled asthma through an evaluation of the prevalence and correlates of uncontrolled asthma among a representative sample of the UK adult population, focusing specifically on patients currently being treated with ICS+LABA. Typically, these patients with moderate to severe asthma may not be adequately controlled with ICS and are prescribed ICS+LABA as maintenance therapy alongside as-needed short-acting β_2_-agonists, and their dosage is tuned depending on asthma control.^2^ In the current study, over 60% of adult patients treated with ICS+LABA reported poorly controlled asthma. Importantly, poor asthma control was associated with a host of negative outcomes—including impaired HRQoL, greater use of healthcare resources and greater work and activity impairment—and substantial direct and indirect costs to society, estimated to be over £6000 million per year (over £1300 million and £4800 million, respectively).

While the primary goals of this study were not focused on assessing adherence, participants’ responses suggested that a significant number of these patients stop taking their medication when they feel better. This requires further investigation, but may point at the need for more detailed patient education, self-management plans and clarification around the importance of medication adherence.

### Interpretation of findings in relation to previously published work

Comparable to our findings, which showed that over 60% of adult patients treated with ICS+LABA reported poorly controlled asthma, previous population-based studies have also found that only a minority of patients report being symptom free, with a similar proportion of individuals as in the current study reporting moderate or poor asthma control.^21, 26, 27^ A structured review of 24 patient surveys conducted in Europe and North America found that patients tolerate poor symptom control, often understate their symptoms, have low expectations of therapy, are unaware of correct drug usage and demonstrate poor adherence to therapy.^28^ Similarly, the REcognise Asthma and LInk to Symptoms and Experience (REALISE) survey conducted in 11 European countries, including UK, found that despite experiencing symptoms and exacerbations, many patients regard their asthma to be controlled.^14^ Moreover, inaccurate assessment of disease severity can result from an inadequate understanding of disease aetiology and poor communication with patients by healthcare providers (HCPs).^28^ In a practice audit for primary care physicians, controlled asthma was positively associated with male sex, age < 35 years and non-smoking or ex-smoking status.^27^ Importantly, and in contrast to previous research,^29^ the current study showed no significant difference in adherence rates between patients with well-controlled and not well-controlled asthma. We acknowledge that assessing adherence using MGLS may overestimate adherence and have poor precision and that this may account for the lack of significant differences between the groups. However, despite these caveats, adherence seemed to be affected by gender, age and income. While males had a lower likelihood of being non-adherent, being older and having a higher income were associated with a higher likelihood of being non-adherent. These findings together suggest that poor asthma control is a result of various factors and improving patients’ understanding and communication with HCPs is important for accurate assessment.

Results of several studies indicate that poor asthma control is associated with decreased quality of life and work productivity and increased healthcare utilisation and costs.^27–35^ Consistent with the results of the current study, results from a cross-sectional study involving 15,149 patients showed that patients with uncontrolled asthma had significantly lower HRQoL as indicated by the Marks Asthma Quality of Life Questionnaire and Paediatric Asthma Caregivers Quality of Life Questionnaire.^30^ Similar results were reported from 2006, 2008 and 2010 European NHWS and 2006 and 2011 US NHWS studies wherein uncontrolled asthma was negatively correlated with patients’ HRQoL assessed by SF-8 and SF-12.^22, 23, 31, 32^ Moreover, uncontrolled asthma had a negative impact on work productivity and activity.

The results of the current study are in agreement with results of previous European NHWS and another US-based survey that reported loss of work productivity and increased use of healthcare resources associated with poor asthma control.^22, 23, 33^ In the present study, healthcare resource utilisation, such as the number of visits to GP, specialist, A&E and hospital, was significantly higher among patients with poorly controlled asthma and increased with comorbidities. An online survey in 11 European countries including 8000 patients found that 23.9% of patients reported visiting an emergency department and 11.7% were hospitalised overnight.^14^ Similarly, a prospective cohort online survey conducted in the US showed a 3-fold greater risk of an asthma-related doctor visit and a 10-fold greater risk of an emergency department visit for asthma in adults with poorly controlled asthma.^34^ Work and activity impairment and greater healthcare utilisation subsequently result in higher medical expenditures. In this study, yearly costs per person were doubled among not well-controlled asthma patients. Such increase in costs due to uncontrolled asthma has also been reported previously.^35^

### Strengths and limitations of this study

A strength of this study is that we analysed a combined sample population of 2 years from the UK NHWS. The NHWS is a large dataset and provides breadth and depth of rigorous patient-reported data with national projections.^36^ It comprises data on the utilisation of healthcare resources and patient-reported outcomes, as well as patient attitudes and approaches to healthcare. This study was therefore able to present comprehensive evidence on work productivity and patient quality of life in patients with asthma.

It is important to consider the results of the current study in the context of the limitations present. An online, panel-based survey has inherent limitations. This was a cross-sectional analysis and therefore causal inferences cannot be drawn between predictor and outcome variables. In addition, although analyses were planned *a priori* and based on the existing literature, if more stringent criteria for multiple analyses were applied, some of the reported pairwise comparisons may become non-significant. However, given the paucity of research in this domain, these results remain important in guiding future research. The data examined in this study were self-reported and may thus have been susceptible to recall errors. Further, responses to questions were not verified with other sources, which may be particularly notable regarding medical diagnoses as it was not possible to confirm them with health records. Finally, asthma-related healthcare utilisation has marked seasonal differences so our derivation of annual rates from 6-month data may result in some imprecision in our estimation of costs.

Although recruitment for the NHWS is designed to ensure representativeness of the UK adult population, the panels from which the sample was drawn may not be truly representative (e.g., over-representation of patients who are younger and in relatively good health) and self-selection of respondents to complete the survey upon being invited may have resulted in a sample of patients whose characteristics differ from those of non-participating respondents (e.g., greater motivation and ability to participate In addition, although a combined sample population of 2 years from the UK NHWS was used, the number of patients analysed was low for some of the subgroup analyses.

Finally, several analyses, while significant, explained a relatively low portion of the overall variance. This suggests that other factors not considered in the current analysis may correlate with the outcome variables, thus reiterating the need for further research.

### Implications for future research, policy and practice

The results of this study, the first of its kind in the UK, emphasise the unmet medical need of patients with asthma who remain symptomatic despite receiving combination therapy with ICS+LABA. Importantly, the costs of this unmet medical need in the UK have been fully quantified for the first time. These estimates are likely to be relevant to areas outside the UK; therefore, these findings will be of interest to the international community. Moreover, it is our opinion that the reported numbers in terms of patients with uncontrolled asthma and yearly costs are of relevance for inclusion in treatment guidelines.

## Conclusions

The current study of UK adult patients with asthma found high rates of poorly controlled disease among respondents. Critically, poor control was associated with impairments across a number of important health outcomes, thus further emphasising the significant burden associated with this medical condition. Further research is needed in order to address the burden of persistent uncontrolled asthma in patients treated with ICS+LABA, as well as contribute to efforts to enhance treatment protocols and improve national asthma management.

## Methods

Direct and indirect costs related to poor asthma control in adult patients treated with ICS+LABA (i.e., ICS and LABA in free or fixed-dose combination) in the UK were estimated as follows: first, the size of the UK adult population taking ICS+LABA was estimated; then, direct costs were estimated by multiplying unit costs by the number of physician visits, hospitalisations and A&E department visits; finally, indirect costs were calculated by applying wage statistics to the work impairment attributed to health problems in employed patients (absenteeism and presenteeism). HRQoL, activity impairment and adherence to asthma prescription medication were also measured, in order to obtain a comprehensive description of the burden associated with poor asthma control among these patients.

### Data source

This study employed a combined sample from the 2010 and 2011 UK NHWS.^36^ The NHWS is a nationally representative online survey of respondents aged 18 years or older conducted by Kantar Health, which gathers data to provide timely patient-reported information on over 160 health conditions, including asthma. This study is part of the European NHWS, which collects data from France, Germany, Italy, Spain and the UK; each nation has the authority to publish country specific data. Data are collected approximately every 18 months in Europe and every 12 months in the United States (US). Before each periodic launch, NHWS gets approved by a US-based Institutional review board (IRB), which in 2010 and 2011 was Essex IRB (Lebanon, NJ).

A random sample within a web-based panel, stratified by gender and age, was included to represent the demographic composition of the UK adult population. The survey sample was recruited from the general population using a web-based consumer panel maintained by Lightspeed GMI.^37^ The consumer panel recruits its panel members through opt-in emails, co-registration with panel partners, e-newsletter campaigns, banner placements and internal and external affiliate networks. All panellists must explicitly agree to be a panel member, register with the panel through a unique e-mail address and complete an in-depth demographic registration profile. These profiles are used to randomly sample panel members for a survey in order to ensure a representative sample. All respondents took part voluntarily and provided informed consent. For the combined 2010–2011 NHWS, 148,171 invitations to participate were sent in the UK; 49,485 potential participants responded; 45,899 were eligible to participate; 5494 were excluded because the quotas for age and gender were reached and 30,065 completed the survey. Further details on panel data management can be found at www.lightspeedgmi.com/global-panels.

### Sample

All respondents from the 2010 and 2011 UK NHWS who self-reported a physician diagnosis of asthma and taking either a fixed-dose or free combination of ICS and LABA for their asthma were included in the analysis. Patients reporting a concomitant physician diagnosis of COPD, chronic bronchitis, or emphysema were excluded. This approach provided a combined sample of more than 500 patients, with approximately half being employed, and allowed for an adequate population size to provide meaningful results. A multivariate analysis was used to adjust for confounders. Sample size calculation was not performed as the primary objective of this study was descriptive.

### Measures

#### Demographics and health characteristics

Several demographic and health characteristic variables were included for description of the study population as well as for consideration as covariates in multivariable models assessing the effect of asthma control on outcomes. These variables included self-reported physician diagnosis of asthma; age; gender; marital status; education; income; employment type; health insurance; body mass index and asthma characteristics such as duration of time with diagnosis, type of diagnosing and prescribing doctor, frequency of asthma conversations with doctor, self-reported severity with and without prescribed asthma medication, frequency of asthma problems, seasonal variation of asthma problems and currently prescribed asthma medications.

#### Comorbidities

An adjusted CCI ref. 38 was calculated by weighting the presence of HIV/AIDS, metastatic tumour, lymphoma, leukaemia, any tumour, moderate or severe renal disease, hemiplegia, diabetes, mild liver disease, ulcer disease, connective tissue disease, chronic pulmonary disease, dementia, cerebrovascular disease, peripheral vascular disease, myocardial infarction and congestive heart failure. The original CCI predicts the likelihood of mortality. In the current study, the CCI was used to obtain an estimate of comorbidity burden, wherein greater total index scores indicate greater comorbidity burden on an individual.

#### Asthma control test™

ACT (QualityMetric Incorporated, Lincoln, RI, USA) is a short, simple, self-reporting tool for identifying patients with poorly controlled asthma.^39^ It measures the elements of asthma control as defined by the National Heart, Lung, and Blood Institute. ACT is an efficient, reliable and valid method for measuring asthma control.^40^ Scores range from 5 to 25, with scores below 20 indicating poor asthma control. Respondents for this study were assigned to the well-controlled group when their ACT score was ≥20 and to the not well-controlled group when their ACT score was <20.

#### Adherence

The MGLS refs. 41, 42 is a generic self-reported medication-taking behaviour scale used for a wide variety of conditions including asthma.^43, 44^ The MMAS-4 consists of four items producing a score ranging from low adherence to high adherence (0–4).^39^

### Outcomes

#### Work productivity and activity impairment

Work productivity was assessed using the WPAI questionnaire, a six-item validated instrument which consists of four metrics: absenteeism (the percentage of work time missed because of one’s health in the past 7 days), presenteeism (the percentage of impairment experienced while at work in the past 7 days because of one’s health), overall work productivity loss (an overall impairment estimate that is a combination of absenteeism and presenteeism) and activity impairment (the percentage of impairment in daily activities because of one’s health in the past 7 days).^45^ Only respondents who reported being full-time, part-time or self-employed provided data for absenteeism, presenteeism and overall work impairment. All respondents provided data for activity impairment.

#### Health-related quality of life

The revised Medical Outcomes Study SF-12v2 Instrument is a multi-purpose, generic instrument comprising 12 questions.^46^ For the purpose of the present analysis, PCS and MCS scores were included. These scores have a mean of 50 and a standard deviation of 10 for the US population. Health state utilities calculated using the short form-6 dimensions algorithm were also included. Utility scores have interval scoring properties and vary from 0 to 1.

#### Use of healthcare resources

Use of healthcare resources was defined by the reported number of visits to traditional healthcare providers (GP or specialist), number of A&E visits and the number of hospitalisations in the past 6 months. All of these variables were also dichotomised to ‘Yes’ (visited a traditional healthcare provider, A&E hospital visit and been hospitalised) versus ‘No’.

#### Cost burden

Cost analysis was conducted at an individual patient level to determine the healthcare costs (both direct and indirect) associated with patients with well-controlled and not well-controlled asthma. To estimate direct costs, the mean costs for an A&E visit, hospitalisation and physician visit were selected. For each respondent, the number of each type of visit was multiplied by 2 to project the annual number of visits and then multiplied by its mean cost provided for 2011 by the National Health Service Department of Health reference cost^47^ and Personal Social Services Research Unit.^48^ Indirect costs were estimated by using annual wage figures for 2011 provided by the Office for National Statistics.^49^ Overall work impairment (from the WPAI questionnaire), which represents the total work time missed or impaired because of either absenteeism or presenteeism, was multiplied by the median wage figure to estimate annual indirect costs.

### Analyses

First, descriptive analysis was used to characterise the population of interest. The 2010–2011 combined NHWS UK sample was projected to the 2011 UK adult population using weights calculated according to UK age and gender data as reflected in the US census international database.^50^

Bivariate analyses were then conducted to test whether there were differences between well- and not well-controlled asthma respondents currently taking ICS+LABA asthma medication regarding demographic variables, general health characteristics and disease variables. Differences between categorical variables were examined using Pearson’s chi-square tests, and differences between continuous variables were examined using *t*-tests. All the comparisons were planned a priori, and all variables significantly different at the 0.05 level were included in the multivariate analyses. Smoking status, age, gender and length of diagnosis were included, regardless of significance, because of their theoretical relevance.

The aim of the multivariate analyses was to determine whether asthma control in patients currently taking ICS+LABA was associated with differences in health outcomes after controlling for potential confounders. Generalised linear model regressions were used to test the association between asthma control and components of HRQoL (MCS, PCS and health utility), work productivity loss (absenteeism, presenteeism, overall work impairment and activity impairment), healthcare resource use (number of GP visits, specialist visits, hospitalisations and A&E visits) and adherence (MGLS score).

To estimate direct costs for cost analysis, the healthcare resource utilisation models were re-run after multiplying the number of visits by the relative unit costs to obtain adjusted estimates. Indirect costs were estimated by re-running the work productivity loss models after multiplying the annual wage figures by the WPAI work impairment measures to obtain adjusted estimates. This method has been outlined in prior research.^51^

## Electronic supplementary material

Supplementary Table 1

Supplementary Table 2

Supplementary Table 3

Supplementary Table 4

Supplementary Table 5
