# Author Correction: The impact of poor asthma control among asthma patients treated with inhaled corticosteroids plus long-acting β_2_-agonists in the United Kingdom: a cross-sectional analysis

**DOI:** 10.1038/s41533-017-0063-5

**Published:** 2017-12-05

**Authors:** Ian D. Pavord, Nicola Mathieson, Anna Scowcroft, Riccardo Pedersini, Gina Isherwood, David Price

**Affiliations:** 1 Respiratory Medicine Unit, Nuffield Department of Medicine, NDM Research Building, Old Road Campus, University of Oxford, Oxford, OX3 7FZ UK; 2 Boehringer Ingelheim Limited, Ellesfield Avenue, Bracknell, Berkshire RG12 8YS UK; 3 Kantar Health, Health Outcomes Practice, 19-31 Church Street, Epsom, Surrey KT17 4PF UK; 4 RTI Health Solutions, Avinguda Diagonal, 605, 9-1, Barcelona, 08028 Spain; 5 Academic Primary Care, University of Aberdeen, Polwarth Building, Foresterhill, Aberdeen, AB25 2ZD UK

**Correction to:** *npj Primary Care Respiratory Medicine* (2017); doi:10.1038/s41533-017-0014-1; Published 09 March 2017

The original version of this article contained errors for which the authors apologize. These errors do not affect the findings of the paper and should not otherwise reduce the veracity or scope of this manuscript and its findings.

After publication, the authors were informed by Amir Goren of Kantar Health that the study did not use the Morisky Medication Adherence Scale (©MMAS-4), but rather the Morisky, Green, and Levine scale (MGLS). All references to MMAS, therefore, have been replaced with MGLS.

Additionally, the authors do not have permission to publish the exact items of the MGLS scale or the scoring algorithm, and therefore cannot make any reference to these items in the paper.

The text, Table 4 and Supplementary Tables 3–5 have been updated accordingly.

