# Supplementary material for: The impact of poor asthma control among asthma patients treated with inhaled corticosteroids plus long-acting β2-agonists in the United Kingdom: a cross-sectional analysis
Source: NPJ Prim Care Respir Med. 2017 Mar 9;27:17. doi: 10.1038/s41533-017-0014-1 (PMC5434793; doi:10.1038/s41533-017-0014-1)
Supplement: Supplementary file 1 — Supplementary Table 1 [file 41533_2017_14_MOESM1_ESM.docx]

Supplementary Table 1. Descriptive statistics for the total 2011 UK and asthma adult populations. The UK population figures are projections from the 2010–2011 UK National Health and Wellness Survey database, while the asthma population figures are projections from the subsample of respondents who self-reported a physician diagnosis of asthma

|  | Males | | Females | | Total | |
| --- | --- | --- | --- | --- | --- | --- |
| Age | **2011 UK population** | **Asthma population** | **2011 UK population** | **Asthma population** | **2011 UK population** | **Asthma population** |
| 18–29 years |  |  |  |  |  |  |
| Unweighted, n | 2509 | 232 | 3371 | 460 | 5880 | 692 |
| Unweighted, % | 16.5 | 17.6 | 22.6 | 25.8 | 19.6 | 22.3 |
| Weighted, n | 5,166,031 | 477,688 | 4,938,515 | 673,900 | 10,104,546 | 1,151,588 |
| Weighted, % | 21.4 | 22.3 | 19.8 | 22.9 | 20.6 | 22.6 |
| 30–39 years |  |  |  |  |  |  |
| Unweighted, n | 2173 | 227 | 2483 | 308 | 4656 | 535 |
| Unweighted, % | 14.3 | 17.2 | 16.7 | 17.3 | 15.5 | 17.2 |
| Weighted, n | 4,109,143 | 429,257 | 3,898,310 | 483,560 | 8,007,453 | 912,817 |
| Weighted, % | 17.0 | 20.0 | 15.6 | 16.4 | 16.3 | 17.9 |
| 40–49 years |  |  |  |  |  |  |
| Unweighted, n | 3035 | 301 | 2968 | 380 | 6003 | 681 |
| Unweighted, % | 20.0 | 22.8 | 19.9 | 21.3 | 20.0 | 21.9 |
| Weighted, n | 4,752,810 | 471,366 | 4,686,472 | 600,020 | 9,439,282 | 1,071,386 |
| Weighted, % | 19.7 | 22.0 | 18.8 | 20.4 | 19.2 | 21.1 |
| 50–59 years |  |  |  |  |  |  |
| Unweighted, n | 2452 | 200 | 2478 | 285 | 4930 | 485 |
| Unweighted, % | 16.2 | 15.2 | 16.6 | 16.0 | 16.4 | 15.6 |
| Weighted, n | 3,540,688 | 288,800 | 4,153,128 | 477,660 | 7,693,816 | 766,460 |
| Weighted, % | 14.7 | 13.5 | 16.6 | 16.2 | 15.7 | 15.1 |
| 60–69 years |  |  |  |  |  |  |
| Unweighted, n | 3408 | 257 | 2637 | 267 | 6045 | 524 |
| Unweighted, % | 22.5 | 19.5 | 17.7 | 15.0 | 20.1 | 16.9 |
| Weighted, n | 4,569,650 | 344,139 | 5,228,173 | 528,781 | 9,797,823 | 872,920 |
| Weighted, % | 18.9 | 16.1 | 20.9 | 17.9 | 19.9 | 17.2 |
| >70 years |  |  |  |  |  |  |
| Unweighted, n | 1593 | 103 | 958 | 85 | 2551 | 188 |
| Unweighted, % | 10.5 | 7.8 | 6.4 | 4.8 | 8.5 | 6.1 |
| Weighted, n | 2,011,959 | 130,089 | 2,066,406 | 183,345 | 4,078,365 | 313,434 |
| Weighted, % | 8.3 | 6.1 | 8.3 | 6.2 | 8.3 | 6.2 |
| Total |  |  |  |  |  |  |
| Unweighted, n | 15,170 | 1320 | 14,895 | 1785 | 30,065 | 3105 |
| Unweighted, % | 100 | 100 | 100 | 100 | 100 | 100 |
| Weighted, n | 24,150,281 | 2,141,339 | 24,971,004 | 2,947,266 | 49,121,285 | 5,088,605 |
| Weighted, % | 100 | 100 | 100 | 100 | 100 | 100 |

Weighted: The estimates were weighted to project to the adult population of the UK and were computed using frequency weights based on sex and age of the UK population aged 18 years and older as described in the International Database of the US Census Bureau.

Unweighted: Based only on the observed sample.
