# Supplementary material for: The impact of poor asthma control among asthma patients treated with inhaled corticosteroids plus long-acting β2-agonists in the United Kingdom: a cross-sectional analysis
Source: NPJ Prim Care Respir Med. 2017 Mar 9;27:17. doi: 10.1038/s41533-017-0014-1 (PMC5434793; doi:10.1038/s41533-017-0014-1)
Supplement: Supplementary file 2 — Supplementary Table 2 [file 41533_2017_14_MOESM2_ESM.docx]

Supplementary Table 2. Descriptive statistics for the total asthma sample treated with ICS+LABA, including all the respondents in the 2010–2011 UK National Health and Wellness Survey database who self-reported to have a prescription for ICS+LABA

| ICS+LABA (free and fixed-dose combination) | | | | | | | |
| --- | --- | --- | --- | --- | --- | --- | --- |
| COPD diagnosis | | **Not well-controlled** | | **Well-controlled** | | **Total** | |
|  |  | **n** | **%** | **n** | **%** | **n** | **%** |
| No | Unweighted | 452 | 74.6 | 249 | 90.9 | 701 | 79.7 |
|  | Weighted | 739,740 | 75.0 | 402,293 | 90.4 | 1,142,033 | 79.8 |
| Yes | Unweighted | 154 | 25.4 | 25 | 9.1 | 179 | 20.3 |
|  | Weighted | 246,012 | 25.0 | 42,589 | 9.6 | 288,601 | 20.2 |
| Total | Unweighted | 606 |  | 274 |  | 880 |  |
|  | Weighted | 985,752 |  | 444,882 |  | 1,430,634 |  |

## COPD, chronic obstructive pulmonary disease; ICS, inhaled corticosteroids; LABA, long-acting β_2_-agonist
