# Supplementary material for: The impact of poor asthma control among asthma patients treated with inhaled corticosteroids plus long-acting β2-agonists in the United Kingdom: a cross-sectional analysis
Source: NPJ Prim Care Respir Med. 2017 Mar 9;27:17. doi: 10.1038/s41533-017-0014-1 (PMC5434793; doi:10.1038/s41533-017-0014-1)
Supplement: Supplementary file 3 — Supplementary Table 3 [file 41533_2017_14_MOESM3_ESM.docx]

Supplementary Table 3. Ordinary least square regression models for health-related quality of life (a) and logistic regression models for adherence (b) within the sample of UK adults treated with ICS+LABA

|  | a. Quality of life (SF-12v2) | | | | | | b. MMAS-4 | | | | | | | | | |  |
| --- | --- | --- | --- | --- | --- | --- | --- | --- | --- | --- | --- | --- | --- | --- | --- | --- | --- |
|  | **MCS** | | **PCS** | | **Health utility** | | **Overall MMAS score** | | **MMAS - Forgetful** | | **MMAS - Careless** | | **MMAS - Feel better** | | **MMAS - Feel worse** | |  |
| **Well-controlled** | 3.8*** | 7.3*** | | 0.1*** | | 1.3 | | 1.1 | | 0.9 | | 0.7* | | 0.6 | |  |  |
|  | (0.90) | | (0.86) | | (0.01) | | (0.22) | | (0.19) | | (0.19) | | (0.13) | | (0.19) | |  |
| **Male** | 1.1 | | 1.9* | | 0.0* | | 0.6** | | 1.4* | | 1.2 | | 1.4 | | 0.9 | |  |
|  | (0.88) | | (0.86) | | (0.01) | | (0.10) | | (0.25) | | (0.23) | | (0.26) | | (0.29) | |  |
| **Age** | 0.2*** | | −0.2*** | | 0.0 | | 1.0*** | | 1.0*** | | 1.0*** | | 1.0** | | 1.0*** | |  |
|  | (0.03) | | (0.03) | | (0.00) | | (0.01) | | (0.01) | | (0.01) | | (0.01) | | (0.01) | |  |
| **Income** | −0.0 | | −0.0 | | −0.0 | | 1.0* | | 1.0 | | 1.0* | | 1.0 | | 1.0 | |  |
|  | (0.02) | | (0.02) | | (0.00) | | (0.00) | | (0.00) | | (0.01) | | (0.01) | | (0.01) | |  |
| **BMI** | | |  | |  | |  | |  | |  | |  | |  | |  |
| Normal range | (ref.) | | (ref.) | | (ref.) | | (ref.) | | (ref.) | | (ref.) | | (ref.) | | (ref.) | |  |
| Underweight | −7.8 | | −0.4 | | −0.1 | | 4.8 | | 0.4 | | 0.8 | | 0.4 | | (dropped) | | |
|  | (5.13) | | (3.82) | | (0.06) | | (3.99) | | (0.32) | | (0.64) | | (0.39) | |  | |  |
| Overweight | −1.9 | | −1.3 | | −0.0 | | 1.2 | | 0.8 | | 0.7 | | 1.2 | | 0.8 | |  |
|  | (1.10) | | (0.98) | | (0.01) | | (0.26) | | (0.18) | | (0.17) | | (0.29) | | (0.30) | |  |
| Obese | −2.0 | | −6.1*** | | −0.1*** | | 0.9 | | 1.1 | | 1.1 | | 1.5 | | 0.8 | |  |
|  | (1.14) | | (1.10) | | (0.01) | | (0.20) | | (0.24) | | (0.26) | | (0.37) | | (0.29) | |  |
| No answer | −3.1 | | −7.4*** | | −0.1** | | 1.5 | | 0.9 | | 0.8 | | 0.7 | | 0.5 | |  |
|  | (1.93) | | (1.89) | | (0.02) | | (0.58) | | (0.36) | | (0.39) | | (0.36) | | (0.43) | |  |
| **Time since diagnosis (years)** | 0.0 | | 0.0 | | 0.0 | | 1.0 | | 1.0 | | 1.0 | | 1.0 | | 1.0 | |  |
|  | (0.03) | | (0.03) | | (0.00) | | (0.01) | | (0.01) | | (0.01) | | (0.01) | | (0.01) | |  |
| **Smoking** | −4.5*** | | −0.8 | | −0.0** | | 1.2 | | 0.8 | | 1.0 | | 1.4 | | 1.0 | |  |
|  | (1.35) | | (1.24) | | (0.01) | | (0.28) | | (0.19) | | (0.25) | | (0.34) | | (0.39) | |  |
| **Charlson Comorbidity Index** | −1.2* | | −3.6*** | | −0.0*** | | 1.2 | | 0.9 | | 1.0 | | 0.8 | | 0.7 | |  |
|  | (0.56) | | (0.65) | | (0.01) | | (0.14) | | (0.12) | | (0.14) | | (0.13) | | (0.23) | |  |
| **MMAS-4 score** | −0.5 | | 0.8* | | 0.0 | |  | |  | |  | |  | |  | |  |
|  | (0.41) | | (0.39) | | (0.01) | |  | |  | |  | |  | |  | |  |
| Constant | 36.5*** | | 53.2*** | | 0.7*** | |  | |  | |  | |  | |  | |  |
|  | (2.03) | | (1.85) | | (0.02) | |  | |  | |  | |  | |  | |  |
| Observations | 697 | | 697 | | 697 | | 697 | | 697 | | 697 | | 697 | | 688 | |  |
| *r*-squared | 0.1 | | 0.3 | | 0.2 | |  | |  | |  | |  | |  | |  |
| LR chi2 |  | |  | |  | | 74.3 | | 49.9 | | 27.4 | | 35.9 | | 23.3 | |  |
| *P* |  | |  | |  | | <0.001 | | <0.001 | | 0.004 | | <0.001 | | 0.009 | |  |
| Models | Ordinary least square regressions | | | | | | Logistic regressions | | | | | | | | | |  |
| **Adjusted values** | **Mean** | |  | |  | | **Probability of non-adherence** | | | | | | | | | |  |
| Not well- controlled | 43.2 | 40.2 | | 0.7 | | 49.8% | | 35.3% | | 22.2% | | 26.6% | | 9.3% | |  |  |
| Well-controlled | 47.1 | 47.5 | | 0.7 | | 55.1% | | 36.5% | | 20.6% | | 19.5% | | 5.7% | |  |  |

## Source: National Health and Wellness Survey combined 2010 and 2011 Robust standard errors in parentheses **P*<0.05; ***P*<0.01; ****P*<0.001 BMI kg/m^2^: Underweight (BMI < 18.5); Normal range (18.5 ≤ BMI < 25); Overweight (25 ≤ BMI < 30); Obese (BMI ≥ 30)

## BMI, body mass index; ICS, inhaled corticosteroids; LABA, long-acting β_2_-agonist; MCS, mental component summary; MMAS-4, four-item Morisky Medication Adherence Scale; PCS, physical component summary; SF-12v2; Medical Outcomes Study 12-Item Short Form Survey Instrument
