# Supplementary material for: The impact of poor asthma control among asthma patients treated with inhaled corticosteroids plus long-acting β2-agonists in the United Kingdom: a cross-sectional analysis
Source: NPJ Prim Care Respir Med. 2017 Mar 9;27:17. doi: 10.1038/s41533-017-0014-1 (PMC5434793; doi:10.1038/s41533-017-0014-1)
Supplement: Supplementary file 4 — Supplementary Table 4 [file 41533_2017_14_MOESM4_ESM.docx]

Supplementary Table 4. Negative binomial regression models for use of healthcare resources (a) and work productivity and activity impairment (b) within the sample of UK adults treated with ICS+LABA

|  | a. Use of healthcare resources, past 6 months | | | | b. Work productivity loss (WPAI) | | | |
| --- | --- | --- | --- | --- | --- | --- | --- | --- |
|  | **Number of GP visits** | **Number of specialist visits** | **Number of A&E department visits** | **Number of hospital visits** | **Absenteeism** | **Presenteeism** | **Overall work impairment** | **Activity impairment** |
| **Well-controlled** | −0.4*** | −0.4*** | −0.4* | −0.6* | −0.5*** | −0.8*** | −0.6*** | −0.7*** |
|  | (0.09) | (0.09) | (0.18) | (0.27) | (0.14) | (0.13) | (0.13) | (0.12) |
| **Male** | −0.1 | −0.2** | −0.1 | −0.2 | −0.3* | −0.4** | −0.3* | −0.4** |
|  | (0.09) | (0.09) | (0.17) | (0.24) | (0.16) | (0.14) | (0.13) | (0.13) |
| **Age** | −0.0 | 0.0 | −0.0 | 0.0 | 0.0*** | −0.0 | 0.0 | 0.0 |
|  | (0.00) | (0.00) | (0.01) | (0.01) | (0.01) | (0.01) | (0.01) | (0.01) |
| **Income** | −0.0 | −0.0 | −0.0 | −0.0 | 0.0*** | 0.0 | 0.0* | 0.0* |
|  | (0.00) | (0.00) | (0.00) | (0.01) | (0.00) | (0.00) | (0.00) | (0.00) |
| **BMI** |  |  |  |  |  |  |  |  |
| Normal range | (ref.) | (ref.) | (ref.) | (ref.) | (ref.) | (ref.) | (ref.) | (ref.) |
| Underweight | 1.0** | 0.9* | 0.9 | 1.0 | −0.6 | −0.2 | −0.3 | −0.3 |
|  | (0.37) | (0.37) | (0.55) | (0.71) | (0.67) | (0.61) | (0.61) | (0.54) |
| Overweight | 0.1 | 0.2 | −0.1 | −0.0 | −0.3 | −0.4* | −0.3 | −0.2 |
|  | (0.12) | (0.12) | (0.21) | (0.30) | (0.20) | (0.16) | (0.16) | (0.15) |
| Obese | 0.2* | 0.4** | 0.3 | −0.2 | −0.7*** | −0.2 | −0.3 | −0.1 |
|  | (0.12) | (0.12) | (0.20) | (0.30) | (0.21) | (0.17) | (0.16) | (0.15) |
| No answer | 0.5* | 0.2 | −0.3 | 0.2 | −1.5*** | 0.2 | −0.3 | −0.2 |
|  | (0.20) | (0.21) | (0.41) | (0.48) | (0.34) | (0.30) | (0.29) | (0.30) |
| **Time since diagnosis (years)** | −0.0* | −0.0 | −0.0 | −0.0 | 0.0 | 0.0 | −0.0 | −0.0 |
|  | (0.00) | (0.00) | (0.01) | (0.01) | (0.01) | (0.01) | (0.01) | (0.01) |
| **Smoking** | 0.1 | −0.1 | 0.3 | 0.3 | 0.1 | 0.2 | 0.1 | 0.0 |
|  | (0.13) | (0.13) | (0.20) | (0.29) | (0.22) | (0.19) | (0.18) | (0.17) |
| **Charlson Comorbidity Index** | 0.24*** | 0.42*** | 0.32*** | 0.32** | −0.01 | −0.10 | −0.11 | −0.00 |
|  | (0.07) | (0.06) | (0.09) | (0.12) | (0.15) | (0.10) | (0.10) | (0.11) |
| **MMAS-4 score** | −0.1 | −0.1* | −0.2** | −0.0 | 0.2*** | −0.0 | 0.1 | 0.1 |
|  | (0.04) | (0.04) | (0.07) | (0.10) | (0.06) | (0.06) | (0.05) | (0.05) |
| Constant | 1.6*** | 1.3*** | −0.1 | −2.3*** | 0.5 | 3.5*** | 3.2*** | 3.3*** |
|  | (0.20) | (0.20) | (0.34) | (0.51) | (0.35) | (0.30) | (0.29) | (0.28) |
| Observations | 697 | 697 | 697 | 697 | 298 | 281 | 298 | 318 |
| Deviance ratio | 0.8 | 1.0 | 0.9 | 0.5 | 4.8 | 3.0 | 3.1 | 2.9 |
| Models | Negative binomial regressions | | | | Negative binomial regressions | | | |
| **Adjusted means** |  |  |  |  |  |  |  |  |
| Not well- controlled | 3.5 | 4.2 | 0.4 | 0.2 | 10.3 | 23.8 | 30.0 | 32.6 |
| Well-controlled | 2.3 | 2.9 | 0.3 | 0.1 | 6.1 | 11.0 | 16.7 | 16.7 |

## Source: National Health and Wellness Survey combined 2010 and 2011 Robust standard errors in parentheses **P*<0.05; ***P*<0.01; ****P*<0.001 BMI kg/m^2^: Underweight (BMI < 18.5); Normal range (18.5 ≤ BMI < 25); Overweight (25 ≤ BMI < 30); Obese (BMI ≥ 30)

## A&E, accident & emergency; BMI, body mass index; GP, general practitioner; ICS, inhaled corticosteroids; LABA, long-acting β_2_-agonist; MMAS-4, four-item Morisky Medication Adherence Scale; WPAI, Work Productivity and Activity Impairment
