# Supplementary material for: The impact of poor asthma control among asthma patients treated with inhaled corticosteroids plus long-acting β2-agonists in the United Kingdom: a cross-sectional analysis
Source: NPJ Prim Care Respir Med. 2017 Mar 9;27:17. doi: 10.1038/s41533-017-0014-1 (PMC5434793; doi:10.1038/s41533-017-0014-1)
Supplement: Supplementary file 5 — Supplementary Table 5 [file 41533_2017_14_MOESM5_ESM.docx]

Supplementary Table 5. Cost analysis: negative binomial regression models for direct (a) and indirect (b) costs within the sample of UK adults treated with ICS+LABA

|  | a. Direct costs (£ per year) | | | | b. Indirect costs (£ per year) | | | |
| --- | --- | --- | --- | --- | --- | --- | --- | --- |
|  | **Physician visits** | **A&E department visits** | **Hospital visits** | **Total direct  costs** | **Absenteeism** | **Presenteeism** | **Overall work impairment** | **Total indirect costs** |
| **Well-controlled** | −0.4*** | −0.5*** | −0.8*** | −0.5*** | −1.0*** | −0.7*** | −0.8*** | −0.8*** |
|  | (0.08) | (0.09) | (0.09) | (0.08) | (0.14) | (0.13) | (0.13) | (0.13) |
| **Male** | −0.2* | −0.1 | −0.0 | −0.2 | 0.0 | −0.3* | −0.5*** | −0.5*** |
|  | (0.08) | (0.08) | (0.10) | (0.08) | (0.15) | (0.14) | (0.13) | (0.13) |
| **Age** | −0.0 | −0.0* | 0.0*** | 0.0 | 0.1*** | −0.0 | 0.0 | 0.0 |
|  | (0.00) | (0.00) | (0.00) | (0.00) | (0.01) | (0.01) | (0.01) | (0.01) |
| **Income** | −0.0 | −0.0 | 0.0 | 0.0 | 0.0*** | 0.0 | 0.0 | 0.0 |
|  | (0.00) | (0.00) | (0.00) | (0.00) | (0.00) | (0.00) | (0.00) | (0.00) |
| **BMI** |  |  |  |  |  |  |  |  |
| Normal range | (ref.) | (ref.) | (ref.) | (ref.) | (ref.) | (ref.) | (ref.) | (ref.) |
| Underweight | 1.0** | 1.3*** | 0.9** | 1.1** | 0.0 | −0.2 | −0.2 | −0.2 |
|  | (0.35) | (0.35) | (0.35) | (0.34) | (0.61) | (0.60) | (0.60) | (0.60) |
| Overweight | 0.1 | −0.2 | 0.3* | 0.1 | −0.5** | −0.4* | −0.4** | −0.4** |
|  | (0.10) | (0.10) | (0.11) | (0.10) | (0.19) | (0.16) | (0.16) | (0.16) |
| Obese | 0.3** | 0.2* | −0.2 | 0.1 | −1.0*** | −0.1 | −0.2 | −0.2 |
|  | (0.10) | (0.11) | (0.11) | (0.10) | (0.21) | (0.16) | (0.16) | (0.16) |
| No answer | 0.4* | −0.5** | −0.1 | 0.1 | −1.9*** | 0.1 | 0.1 | 0.1 |
|  | (0.18) | (0.19) | (0.19) | (0.18) | (0.32) | (0.30) | (0.30) | (0.30) |
| **Time since diagnosis (years)** | −0.0 | −0.0*** | −0.0** | −0.0* | 0.0 | 0.0 | 0.0 | 0.0 |
|  | (0.00) | (0.00) | (0.00) | (0.00) | (0.01) | (0.01) | (0.01) | (0.01) |
| **Smoking** | −0.0 | 0.5*** | 0.3** | 0.2 | −0.3 | 0.3 | 0.2 | 0.2 |
|  | (0.11) | (0.11) | (0.12) | (0.11) | (0.21) | (0.18) | (0.18) | (0.18) |
| **Charlson Comorbidity Index** | 0.35*** | 0.40*** | 0.55*** | 0.41*** | −0.24 | −0.15 | −0.12 | −0.12 |
|  | (0.06) | (0.07) | (0.07) | (0.06) | (0.16) | (0.09) | (0.09) | (0.09) |
| **MMAS-4 score** | −0.1* | −0.2*** | 0.0 | −0.0 | 0.3*** | −0.0 | 0.0 | 0.0 |
|  | (0.03) | (0.04) | (0.04) | (0.03) | (0.06) | (0.05) | (0.05) | (0.05) |
| Constant | 6.3*** | 5.0*** | 5.6*** | 6.8*** | 4.7*** | 8.8*** | 8.7*** | 9.4*** |
|  | (0.17) | (0.16) | (0.16) | (0.16) | (0.34) | (0.29) | (0.29) | (0.29) |
| Observations | 697 | 697 | 697 | 697 | 306 | 281 | 281 | 281 |
| Deviance ratio | 1.4 | 8.0 | 12.3 | 2.2 | 13.7 | 7.8 | 7.8 | 8.4 |
| Models | Negative binomial regressions | | | | Negative binomial regressions | | | |
| **Adjusted means (£)** | | | | | | | | |
| Not well-controlled | 551.0 | 95.5 | 708.4 | 1,271.3 | 2,746.9 | 4,480.0 | 5,237.6 | 10,475.4 |
| Well-controlled | 375.0 | 60.2 | 322.5 | 750.8 | 1,012.3 | 2,180.9 | 2,462.7 | 4,925.2 |

## Source: National Health and Wellness Survey combined 2010 and 2011 Robust standard errors in parentheses **P*<0.05; ***P*<0.01; ****P*<0.001 BMI: Underweight (BMI < 18.5); Normal range (18.5 ≤ BMI < 25); Overweight (25 ≤ BMI < 30); Obese (BMI ≥ 30)

## A&E, accident & emergency; BMI, body mass index; ICS, inhaled corticosteroids; LABA, long-acting β_2_-agonist; MMAS-4, four-item Morisky Medication Adherence Scale
